# Minor salivary gland stem cells: a comparative study of the biological properties under clinical-grade culture conditions

**DOI:** 10.1007/s00441-023-03789-z

**Published:** 2023-05-30

**Authors:** Dimitrios Andreadis, Ioannis Angelopoulos, Elena Aggelidou, Evangelia Gousopoulou, Joachim Volk, Athanasios Poulopoulos, Aristeidis Kritis, Werner Geurtsen, Athina Bakopoulou

**Affiliations:** 1 Department of Oral Medicine/Pathology, School of Dentistry, Faculty of Health Sciences, Aristotle University of Thessaloniki, Thessaloniki, Greece; 2 Department of Prosthodontics, Tissue Engineering Core Unit, School of Dentistry, Faculty of Health Sciences, Aristotle University of Thessaloniki, Thessaloniki, Greece; 3 Department of Physiology and Pharmacology, School of Medicine, Faculty of Health Sciences, Aristotle University of Thessaloniki, Thessaloniki, Greece; 4 cGMP Regenerative Medicine Facility, Department of Physiology and Pharmacology, School of Medicine, Faculty of Health Sciences, Aristotle University of Thessaloniki, Thessaloniki, Greece; 5 Department of Conservative Dentistry, Periodontology and Preventive Dentistry, Hannover Medical School (MHH), Hannover, Germany

**Keywords:** Minor salivary gland stem cells (mSG-SCs), Clinical-grade expansion, Good manufacturing practice compliant cell preparation, Prolonged culture, “Stemness” properties

## Abstract

Development of clinical-grade, cell preparations is central to cGMP (good manufacturing practice compliant) conditions. This study aimed to investigate the potential of two serum/xeno-free, cGMP (StemPro, StemMacs) culture media to maintain “stemness” of human minor salivary gland stem cell (mSG-SC) cultures compared to a complete culture medium (CCM). Overall, StemMacs resulted in higher proliferation rates after p.6 compared to the conventional serum-based medium, while StemPro showed substantial delays in cell proliferation after p.9. The mSG-SCs cultures exhibited two distinct cell populations at early passages a mesenchymal subpopulation and an epithelial-like subpopulation. Expression of several markers (CD146, STRO-1, SSEA-4, CD105, CD106, CD34, K 7/8, K14, K18) variably decreased with prolonged passaging (all three media). The percentage of SA-β-gal positive cells was initially higher for StemMacs compared to StemPro/CCM and increased with prolonged passaging in all cases. The telomere fragment length decreased with prolonged passaging in all three media but more pronouncedly for the CCM. Expansion under serum-free conditions caused pronounced upregulation of ALP and BMP-2, with parallel complete elimination of the baseline expressions of LPL (all three media) and ACAN (serum-free media), therefore, showing a preferential shift of the mSG-SCs towards osteogenic phenotypes. Finally, several markers (Nanog, SOX-2, PDX-1, OTX2, GSC, HCG) decreased with prolonged culture, indicating successive loss of “stemness”. Based on the findings, it seems that StemPro preserve stemness of the mSG-SCs after prolonged culture. Nevertheless, there is still a vacant role for the ideal development of clinical-grade culture conditions.

## Introduction

In recent years, there has been strong interest in the use of salivary gland stem cells in Regenerative Medicine (RM). A search in the *clinicaltrials.gov* (date 17/3/22) registry revealed 21 results for clinical trials employing salivary gland stem cells mainly derived from major salivary glands. The rationale for the clinical application of salivary gland stem cells includes the regeneration of the salivary gland parenchyma in cases of severe or irreversible damage, caused by several pathologies or conditions. For instance, these include the therapeutic radiation of the head and neck area; drug-induced xerostomia; the presence of Sjögren’s syndrome or other autoimmune sieladenitis; metabolic diseases, such as diabetes mellitus; or surgical removal of the salivary glands in case of neoplasms. All these conditions are associated with salivary gland dysfunction, leading to xerostomia, which affects a large portion of elderly individuals. Oral dryness (another term for xerostomia) leads to dysphagia, difficulties in speaking, increase of the prevalence of dental caries and periodontitis, decreased resistance to infections (bacterial, viral fungal), and loss of dentures cohesion, and overall, it compromises the patients’ quality of life-QoL (Rotter et al. 2008; Rotter et al. 2010; Feng et al. 2009; Cha 2017).

Specifically, minor salivary gland stem cells (mSG-SC) have been used in different mouse models, including transplantation of the cell populations (cell-based therapy) or transplantation of salivary organoids (cKIT^+^ salispheres) in radiation-damaged salivary glands, which resulted in the development of amylase-producing tissues and partial regeneration of the gland (Lombaert et al. 2008). Besides, the possibility of autologous transplantation of salivary gland with in vitro regenerative techniques from cells of the same organism in an experimental mouse model resulting in saliva production has been also reported (Tanaka et al. 2018).

Culture media used for cell expansion and maintenance play a key role in the biological properties of stem cells, such as stemness and differentiation capacity, especially for clinical applications (Liu et al. 2017). Biological agents containing cells intended for clinical application (advanced therapy medical products (ATMPs)) need to fulfill certain regulatory criteria recommended by international organizations, such as the European Union directives. In addition, the maintenance of their stem cell phenotypic characteristics and their genetic material stability (especially maximum potency for tissue regeneration with the correct number of population doublings) is crucial (Schellenberg et al. 2012). Despite its contribution to cellular proliferation, the supplementation of animal serum in the culture medium is related to various complications, such as heterogeneity of the cellular population (Shahdadfar et al. 2005). Also, the proteins contained in the animal serum may cause biological complications to the patients receiving autologous stem cells cultured in such serum-containing media (possible autoimmune or immune reactions or transfer of zoonoses to humans) (Shahdadfar et al. 2005). Unfortunately, any replacement of bovine serum with non-human serum or human platelet solution presents technical difficulties and reduces cell proliferation in large scale. Recently, new types of media for cellular culture that have no animal derivatives (serum/xeno free culture media) have begun to appear aligned with international instructions for cGMP in different stem cell cultures (van der Valk et al. 2018; Karnieli et al. 2017; Hamada et al. 2020).

This study followed the notion of previous comparison studies between different types of media with or without serum evaluating the proliferation rate, multilineage differentiation capacity, morphological and immunophenotypic characteristics, and cellular aging after a prolonged incubation of stem cells (Bakopoulou et al. 2017). More analytically, in this study, we have evaluated the prolonged incubation of mSG-SC of oral mucosa in three different culture media and two serum-free that comply with cGMP quality conditions (StemPro and StemMacs) compared to a conventional bovine serum containing culture medium (Life Technologies, USA). The objective was to investigate the capacity of culture media without the supplementation of animal bovine serum- in accordance with standards for cGMP cell culture development- to yield enough cells which survive, proliferate, and maintain stemness. This study design assists, offering a good clinical application alternative for the growth of stem cells of epithelial/mesenchymal origin from glandular tissues that may be used in critical tissue therapies including not only salivary tissue regeneration (homologous) but also for heterologous tissue application such as pancreatic. In addition, the scientific motivation for this study was the fact that different tissue culture conditioned media may adversely affect the phenotypic and functional characteristics as well as stem cell activities. These may include the reduced proliferation ratio, aging (cellular senescence), immunophenotypic and molecular changes, and reduced differentiation capacity. In turn, these altered characteristics may lead to improper and effective applications with unpredictable pathologic consequences.

## Material and methods

### Isolation of human minor salivary glands biopsies

Human minor salivary glands were isolated from lower lip biopsies taken for Sjögren’s syndrome investigation, as described previously (Andreadis et al. 2014). These biopsies (*n* = 3) were microscopically negative for Sjögren’s syndrome or other type of sialadenitis. The study and all the experimental methodology were performed according to Helsinki Declaration II and were approved by ethical committee of the School of Dentistry, Aristotle University of Thessaloniki, Greece (reference number 322/15–4-2013).

### Establishment of minor salivary gland stem cell culture (mSG-SC)

Minor salivary gland stem cells (mSGSCs) were isolated from biopsies of minor salivary glands of lower lip from patients aged between 22 and 67 years old. All healthy individuals were non-smokers and systemically healthy. After clinical and laboratory evaluation, they were confirmed negative for Sjögren’s syndrome. The process of isolating mSGSCs was carried out by means of the enzymatic separation, as already described previously (Andreadis et al. 2014). Specifically, minor salivary glands were dissected with a scalpel in small Sects. (1–2 mm^2^) and immersed in a solution with yeast collagenase I (3 mg/ml) and dispase (dispase) II (4 mg/ml) (Life Technologies, USA) following incubation at 37 °C for 1 h with occasional agitation to achieve the breakdown of individual cells. Later, cells were placed in 25-cm^2^ flasks (p.0) and incubated at 37 °C in 5% CO_2_ when cells reach 70–80% confluency and were transferred to 75 cm^2^ flasks for further subculturing. The medium was changed every other day, and the cells were used until passage 10. For salivary stem cell expansion, 3 different culture media were used (CCM vs StemMacs vs StemPro): specifically, (1) α-MEM (Life Technologies, USA) supplemented with 15% FBS (Life Technologies, USA), 100 μM ascorbic acid phosphate (Sigma-Aldrich, Germany) and 1% antimicrobial solution (100 units/ml penicillin, 100 mg/ml streptomycin) (Life Technologies, USA). The cells were detached using 0.25% trypsin/1 mM solution EDTA for 5 min at 37 °C and replated at a density of 5000 cells/cm^2^. (2) StemPro MSC SFM XenoFree (“StemPro”, Life Technologies, USA) was prepared according to the manufacturer instructions; 98 ml StemPro MSC SFM basic medium was supplemented with 1 ml of StemPro supplement, 1 ml L-glutamine solution GlutaMAX – I CTS, and 50 μl gentamicin solution (50 mg/ml). Plastic surfaces were coated with CELLstart CTS (Life Technologies, USA). Cells were detached with TrypLE™ Select CTS™ (Life Technologies, USA) as an alternative to porcine trypsin used elsewhere for 4–5 min at 37 °C and replated at a density of 5000 cells/cm^2^. (3) Miltenyi StemMacs MSC expansion media kit XF (“StemMacs”; Miltenyi Biotech GmbH, Germany) was prepared according to the manufacturer instructions; 98.6 ml of StemMacs MSC basal expansion media XF was supplemented with 1.4 ml StemMacs MSC expansion media XF supplement and 50 μl gentamicin (50 mg/ml). For cell detachment and further subculturing, TrypLE™ Select CTS™ was used as mentioned previously for 5 min at 37 °C, and cells were seeded at a density of 5000 cells/cm^2^.

### Immunophenotypic characterization of mSG-SCs with flow cytometry

The mSGSCs were characterized based on the 3 different culture media for mesenchymal, endothelial, fetal, and hematopoietic cell membrane markers of stem cells, such as CD90/Thy-1, CD73, CD146/MUC18, STRO-1, CD105, CD106, CD34, and the embryonic marker SSEA-4, as well as glandular epithelial cell markers (cytokeratins 7/8, 14, 18) by flow cytometry as described previously (Andreadis et al. 2014). For flow cytometry, the following fluorochrome conjugated mouse anti-human antibodies were used: CD90/Τhy-1-FITC, CD73-PE, CD146/MUC18-PE, STRO-1-FITC, CD105/endoglin-APC, CD106/VCAM-1-APC, CD34-APC, SSEA-4-FITC, cytokeratins 7/8-ΡΕ, cytokeratin 14-FITC, and cytokeratin 18-FITC (all BioLegend, Fell, Germany). Analysis was performed with the Guava^®^ cytometer easyCyte 8HT benchtop flow cytometer (Merck Millipore, USA). A total of 50,000 events per sample were recorded, and data were analyzed with the software GuavaSoft 3.1.1 and Summit 5.1. In addition to determining the percentage of cells positive for each marker, the cell size and cell internal complexity (granularity) distribution profiles were analyzed by forward scatter (FSC) vs side scatter (SSC) fluorescence intensity plots, respectively.

### Evaluation of morphological characteristics and cell proliferation

Cell morphology was studied in reverse phase contrast view finder (Zeiss Axiovert 40; Carl Zeiss microimaging, GmbH, Göttingen, Germany) equipped with digital camera and an analysis software (Carl Zeiss Axiovision 4.6 software). Images were taken from selected areas, representative of the cell growth in cell culture. The proliferation potential of mSG-SCs was evaluated by measuring the population doubling time (PDT) over passages based on the following formula: 2_*n*_ = Nx / No, where *Nx* was the number of cells measured by hemocytometer after their detachment and *No* was the number of cells that were originally plated. Population doubling (PD) was calculated as PD = log_2_ (Nx / No). At p.0, the total number of cells initially attached in the flask after 48 h were defined as No. Lastly, PDT for each passage was estimated as *t*/*n*, where *t* stands for time and *n* was the number of PD through passage.

### Assessment of mSG-SC senescence

#### Senescence-related β-galactosidase assay

The expression of senescence was measured based on expression of β-galactosidase (SA-β-gal activity) at different representative passages (p.2–3), (p.6–7), and (p.10–11) using a beta galactosidase chromogenic assay kit (Sigma-Aldrich). Cells were fixed with 4% PFA, washed with PBS and incubated with staining β-galactosidase (40 mM citric acid sodium phosphate buffer, 1 M NaCl, 5 mM ferrocyanide, 5 mM ferricyanide, 2% DMF, 20 mM MgCl_2_, X-GAL 1 mg/ml in DMSO) for 14–16 h at 37 °C. Positive (blue stained) and negative cells were measured using a light microscope in random positions, and the total percentage of positive cells was evaluated.

#### Evaluation of mSG-SC telomere length measurement

Total genomic DNA (gDNA) was isolated from cells with the Nucleospin tissue DNA isolation kit (Macherey Nagel, Germany). The relevant telomeres’ length in different cells was measured with the TeloTAGGG telomere length assay kit (Roche, USA) according to manufacturer instructions; specifically, 2 μg gDNA/sample was initially digested with the Hingl/RSA1 system, then electrophoresed in 1% agarose/TAE gel, and transferred for southern blot on positively charged nylon membrane at 20 X potassium citrate buffer. The gDNA hybrid was tagged with a non-isotopic marker labeled with digoxygen (DIG) incubated with an antibody against DIG covalently linked to alkaline phosphatase and visualized with an optical analysis system chemiluminescence (ECL) (MicroChemi, Israel). The length of the telomeres was evaluated with final restriction fragment analysis (TRFs, terminal restriction fragments) calculated as follows: TRFs = Σ (ODi) / Σ (ODi / Li), where *ODi* is the intensity of the chemiluminescent signal and *Li* was the length of the TRF at position *i*. TRF analysis was done with a special software TeloTool (Göhring et al. 2014).

#### Evaluation of proteomic progenitor stem cell marker expression

The protein expression of several markers pluripotency (Oct-3/4, Nanog, SOX2, E-Cadherin, α-Fetoprotein-AFP, GATA-4, HNF-3β/FoxA2, PDX-1/IPF1, SOX17, Otx2, TP63/TP73L, Goosecoid-GSC, Snail, VEGF R2/KDR/Flk-1, HCG) was investigated with the Proteome Profiler array: human pluripotent stem cell array kit (R&D Systems Inc., Europe Ltd., UK), after long-term culture in the serum-containing medium (CCM), as this medium showed the most pronounced effect in reducing the telomere length of the mSG-SCs.

Briefly, the nitrocellulose membranes incubated with different antibodies were further incubated with a blocking buffer solution for 1 h at room temperature; cell lysates were placed in contact the membranes and further incubated at 2–8 °C in a stirring platform overnight. The following day, the membranes were washed and further incubated with a streptavidin-HRP secondary antibody for 2 h in a stirring platform. Then, a chemiluminescent visualization reagent (Chemi Reagent Mix) was applied to the membrane. Finally, the membrane was visualized using an optical chemiluminescence analysis (ECL) system (MicroChemi; DNR Bio-Imaging Systems Ltd., Neve Yamin, Israel). Image J software (Image J. US National Institutes of Health, Bethesda) combined with the dot blot micromodule tool was used to quantify positive expression of the membrane.

#### Assessment of mRNA expression of osteogenic, adipogenic, and chondrogenic markers

The upregulation/downregulation of different osteogenic (alkaline phosphatase, ALP; bone morphogenetic protein 2, BMP-2), chondrogenic (aggrecan-ACAN, transcription factor SOX-9), and adipogenic (lipoprotein lipase-LPL, peroxisome proliferator-activated receptor-gamma-PPAR-γ) markers were evaluated with qPCR. The primers of these genes are summarized in Table 1. Specifically, total mRNA was isolated from mSG-SCs at p.2–3, p.6–7, and p.10-p.11 passages using Nucleospin RNA isolation kit, (Macherey Nagel, Germany) following reversed transcription in cDNA (1 μg/sample) using the superscript first-strand synthesis kit (Invitrogen). qPCR reactions were performed with the SYBR-Select PCR Master Mix in a Step One Plus thermal cycler (all Applied Biosystems, USA). The reaction included a step 50 °C for 2 min for uracil-N-glycosylase (UNG), at 95 °C for 2 min for the activation of AmpliTaq DNA polymerase. Then, 40 qPCR cycles were followed, containing a denaturation step (denaturation) for 15 s at 95 °C, and annealed annealing/extension of DNA strands for 1 min at 60 °C. The results were calculated according to the performance (amplification efficiency) with appropriate application (LinRegPCR) and normalized against 3 housekeeping genes: (1) β2-microglobulin (B2M) and (2) succinate dehydrogenase (SDHA) were used for the investigation of lineage-specific markers, while the (3) YWHA2 was additionally used for the experiments investigating osteogenic and chondrogenic differentiation after the induction with selective media. The selection of the housekeeping genes was based on geNorm analysis indicating the two most stable for each type of experiment (Vandesompele et al. 2002).

**Table 1:** Gene primers designed for real-time PCR and the individual amplicon sizes of their products

**Gene**	**Type of marker**	**Forward (5′–3′)**	**Reverse (5′–3′)**	**Amplicon size (bp)**
BMP-2	Osteogenic	GGAACGGACATTCGGTCCTT	AGTCCGTCTAAGAAGCACGC	100
ALP	Osteogenic	CCGTGGCAACTCTATCTTTGG	CAGGCCCATTGCCATACAG	89
RUNX2	Osteogenic	CCACCGAGACCAACAGAGTC	TCACTGTGCTGAAGAGGCTG	118
BGLAP	Osteogenic	GACTGTGACGAGTTGGCTGA	AAGAGGAAAGAAGGGTGCCT	137
ACAN	Chondrogenic	CACCTCCCCAACAGATGCTT	GGTACTTGTTCCAGCCCTCC	107
SOX9	Chondrogenic	AGGAAGTCGGTGAAGAACGG	CGCCTTGAAGATGGCGTTG	84
PPAR-γ	Adipogenic	GACAACCTGCTACAAGCCCT	TTGGCAAACAGCTGTGAGGA	71
LPL	Adipogenic	CGAGCGCTCCATTCATCTCT	CCAGATTGTTGCAGCGGTTC	137
B2M	Housekeeping	TGTCTTTCAGCAAGGACTGGT	ACATGTCTCGATCCCACTTAAC	138
SDHA	Housekeeping	GCATGCCAGGGAAGACTACA	GCCAACGTCCACATAGGACA	127
YWHAZ	Housekeeping	GCTCTCGATTGGAACGCCT	TCCATGACTGGATGTTCTGCT	125

#### Evaluation of the in vitro osteogenic and chondrogenic differentiation potential

mSG-SCs were induced with 3 different culture media (CCM vs StemMacs vs Stem-Pro) towards osteogenic, chondrogenic, and adipogenic phenotype to evaluate their differentiation capacity at p.2–3, p.6–7, and p.10–11. For osteogenic and chondrogenic differentiation, 300,000 cells were seeded in 6 well plates until reaching confluency, and the medium was changed (StemPro osteogenic and StemPro chondrogenic differentiation kits; Life Technologies). After 14 days of continuous culture, the RNA of the cells was isolated to perform qPCR as described in previous section, and the expression of different osteogenic markers (ALP, BMP-2, bone gamma-carboxyglutamate protein/osteocalcin (BGLAP), and chondrogenic (ACAN, SOX-9)) was analyzed. Furthermore, mSG-SCs were evaluated for bone formation potential. Therefore, the wells were exposed to osteogenic medium for 21 days and further were evaluated by histochemical staining with 1% red of Alizarin (Alizarin Red S) followed by measurement of mineralized tissue by its dissolution coupled staining with cetyl pyridinium chloride (CPC), as already described previously (Andreadis et al. 2014). The optical density (OD) was measured at 550 nm with an ELISA spectrophotometer (Epock; Biotek Instruments, USA).

### Statistics

All experiments were performed in 2–4 technical replicates and 3 biological replicates for each donor, except the qPCR assays that were performed as above, but using cells pooled from the 3 donors. Statistical analysis was held one-way analysis of variance (one-way or two-way ANOVA) with multiple comparisons between the different parameters such as 3 different types of cell culture media (CCM vs. StemMacs vs. StemPro) and early vs middle vs late passages. Tukey’s post-hoc test was used for correlations with Prism 6.0 software (GraphPad, CA, USA). The normal distribution was confirmed by the D’Agostino and Pearson tests. The data were expressed in mean values ​​with constant deviations (SD, standard deviation).

## Results

### Estimation of cell proliferation and morphological features of mSG-SCs

**Fig. 1:**
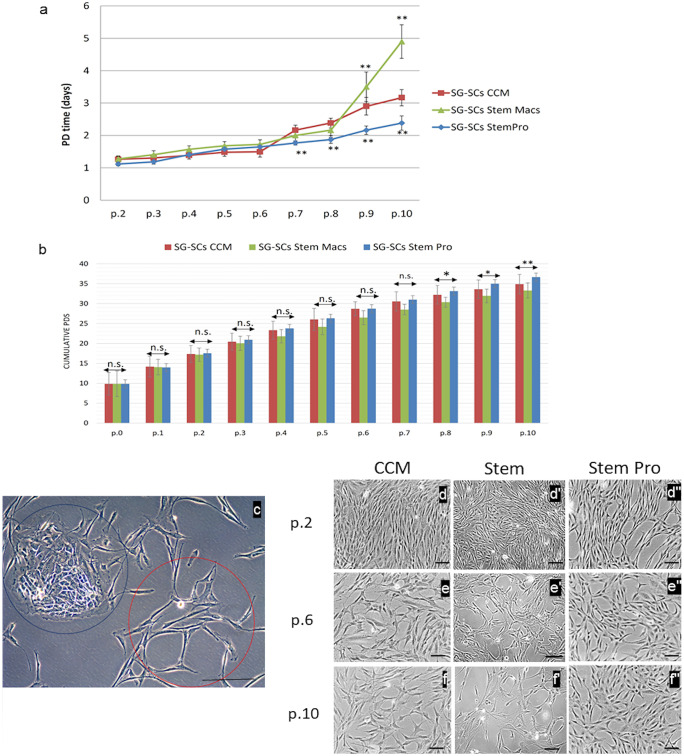
Growth dynamics—PD time and cumulative PDs. **a** PDT replication time (in days) of the mSG-SCs for each culture medium from p.2 to p.10. PDT in days is the mean (± constant deviation, SD) for mSG-SCs (*n* = 3 donors/culture medium), in experiments repeated three times (in two repetitions for each). **b** Total number of duplications (PDs) (mean ± constant deviation, SD) for mSG-SCs (*n* = 3/culture medium). Calculations based on proportion of the number of cells at the beginning and end of each re-culture in experiments repeated three times (in two technical copies for each). The statistical analysis correlation between the passages and the three-culture media was performed with two-way ANOVA and Tukey’s post-hoc test for the analysis of the means of the individual parameters. An asterisk indicates the statistically significant increase of PDT in the successive passages of each culture medium (*p < 0.05, **p < 0.01). **c** The two distinct cell populations: an ovoid-epithelial type cells (blue circle) and the mesenchymal type elongated, spindle-shaped cells of various sizes and with multiple cytoplasmic invasions (red circle). **d**–**f**'' microscope photographs show cell populations in CCM/StemMacs/StemPro and more specifically in passages p.2, p.6, and p.10 with phase contrast microscope (scale: 100 μm)

Primary mSG-SCs from minor salivary glands were cultured in the three different media (CCM, StemPro, StemMacs), and their proliferation was evaluated in different passages from 1 to 10. A first key parameter evaluated is the population doubling time (PDT) of cell cultures. PDT was calculated by measuring the number of cells at the beginning and end of each passage. The time required from the initial phase of mSG-SCs culture after the enzymatic digestion step to p.1 was 8.9 (± 2.3), 9.3 (± 3.2), and 9.1 (± 1.4) days for culture of mSG-SCs in CCM, StemMacs, and StemPro, respectively. This suggests a similar growth potential of mSG-SCs at the initial stage. fA shows the PDT replication time (in days) of the mSG-SCs for each culture media from p.2 to p.10. In Fig. 1a, the cell proliferation rate of mSG-SCs after subsequent cultures (p.2–p.10) in all three different culture media is summarized.

PDT in days is the mean for mSG-SCs (*n* = 3 donors/culture medium), in experiments repeated three times (two technical replicates). According to Fig. 1a, PDT is similar and remains stable with a slight increase in proliferation rate of mSG-SCs and with small differences between all three-culture media until p.6. However, from p.7 onwards, StemPro had a statistically significantly lower PDT compared to CCM (*p* < 0.01), whereas from p.9 onwards, StemMacs had a statistically significantly greater PDT compared to CCM (*p* < 0.01). This observation indicates that the mSG-SCs in StemMacs retain their proliferation capacity even after many subcultures compared to CCM; in contrast to StemPro, a significant downregulation of the cell proliferation rate indicates the possible loss of the stemness and the onset of cellular aging. Figure 1b depicts the total number of PDs of mSG-SCs cultures for each of the three-culture media from the beginning of the culture in p.0 up to p.10. As for the total number of PDs in mSG-SCs, this increases as the subculturing is ongoing, with no statistically significant differences between all three-culture media up to p.7. Statistically significant differences (*p* < 0.05) were observed in p.8, p.9, and p.10 between StemMacs and StemPro, with the latter achieving a greater number of replication doublings. Overall, the total number of PDs after ten subcultures (passages) was 33.9 (± 2.4) for CCM, 32.7 (± 1.9) for StemMacs, and 35.9 (± 1.2) for StemPro. After the completion of the first two subcultures (p.2), the total number of cells were approximately 15–20 million, and after three subcultures, 4–60 million (acceptable number for clinical applications).

Morphological characteristics of cells in the cultures with all three different media are evaluated with a phase contrast microscope, as shown in Fig. 1c and Fig. 1(d–f''). It was observed that in p.0 in all three different media, there were two distinct cell populations, for example, on the one hand ovoid cells (blue circle) and on the other elongated, spindle-shaped cells of various sizes with multiple cytoplasmic invasions (red circle). Similar findings were observed previously (Andreadis et al. 2014). This observation was more pronounced with the CCM. Later, with further subculturing (p.1–2), an additional reduction of the spheroidal-ovoid cells was observed, and from p.3 onwards, it was evident in all three-culture media the presence of almost exclusively spindle-shaped cells (i.e., elimination of the epithelial cells), which developed multiple cellular connections between them, giving the idea of a mesh image (especially in StemMacs).

### Immunophenotypic characterization of mSG-SCs by flow cytometry

**Fig. 2:**
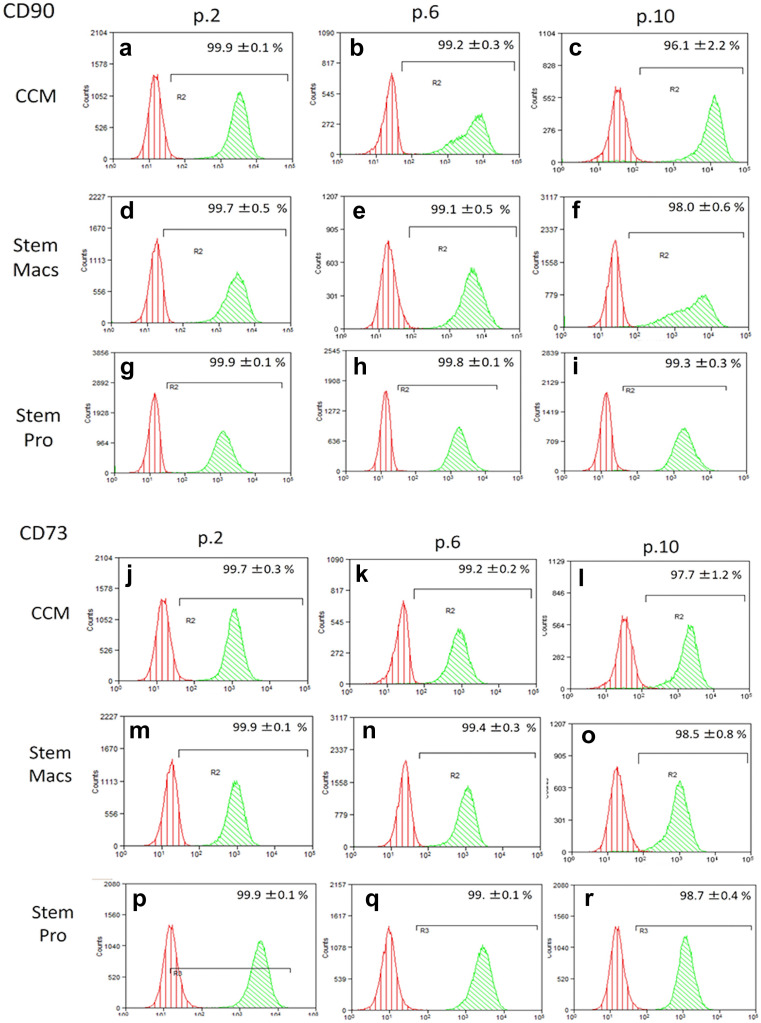
Flow cytometric analysis of **a**–**i** CD90/Thy-1 and **j**–**r** CD73 for each expansion medium at early, middle, and late passages. Values are mean (± SD) of three independent experiments of a single representative mSG-SCs

**Fig. 3:**
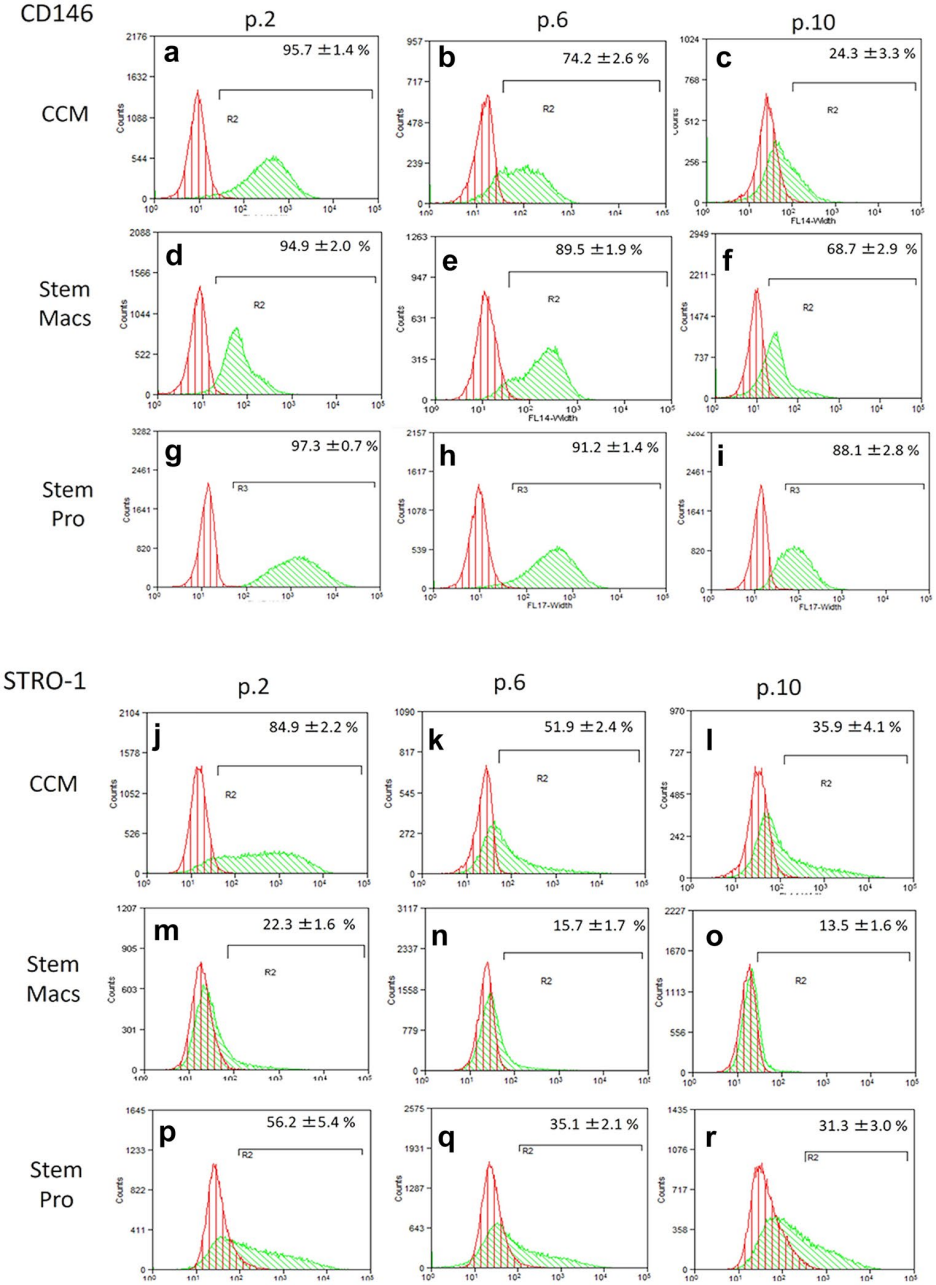
Flow cytometric analysis of **a**–**i** CD146/MUC18 and **j**–**r** STRO-1 for each expansion medium at early, middle, and late passages. Values are mean (± SD) of three independent experiments of a single representative mSG-SCs

**Fig. 4:**
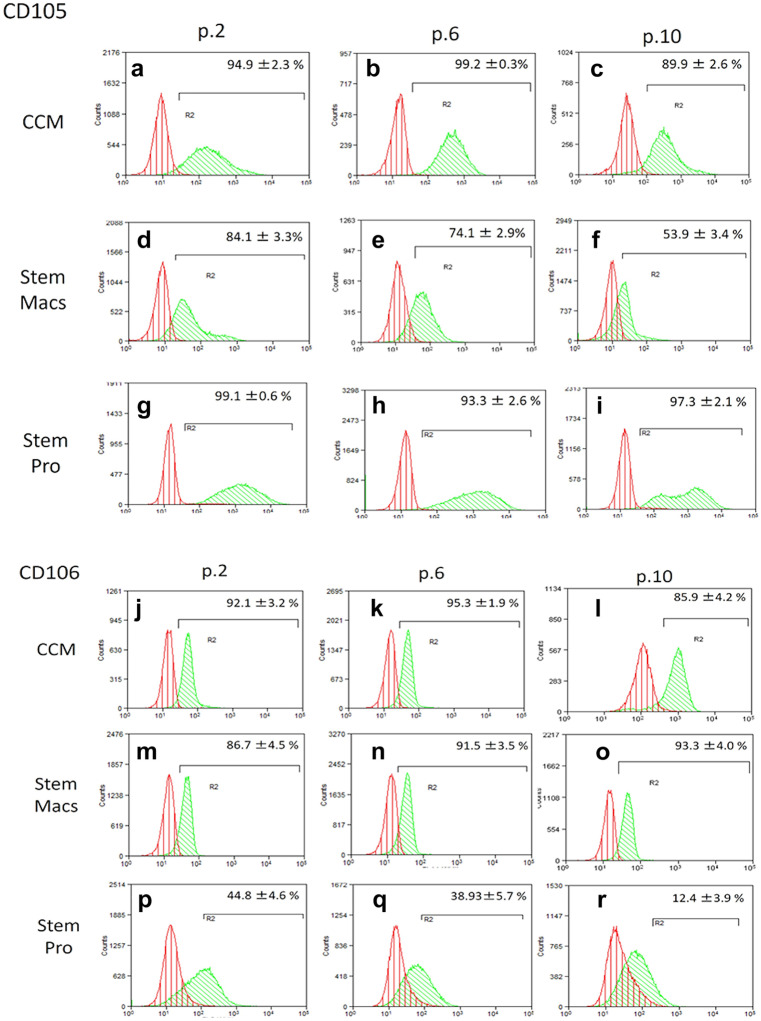
Flow cytometric analysis of **a**–**i** CD105 and **j**–**r** CD106 for each expansion medium at early, middle, and late passages. Values are mean (± SD) of three independent experiments of a single representative mSG-SCs

**Fig. 5:**
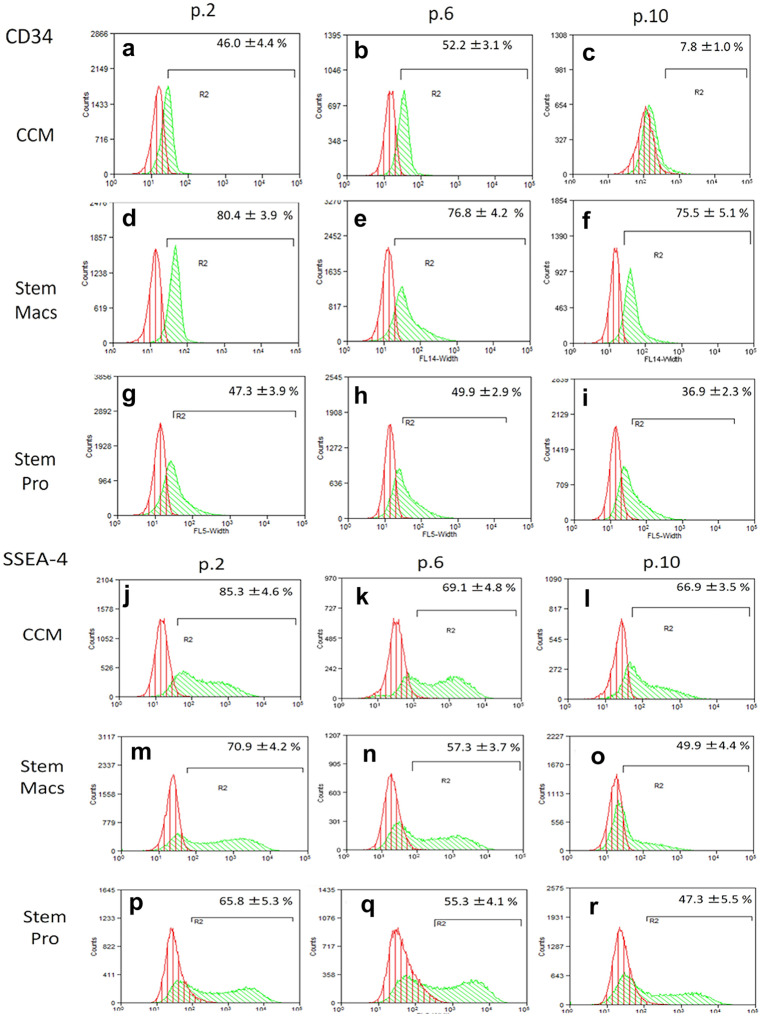
Flow cytometric analysis of **a**–**i** CD34 and **j**–**r** SSEA-4 for each expansion medium at early, middle, and late passages. Values are mean (± SD) of three independent experiments of a single representative mSG-SCs

**Fig. 6:**
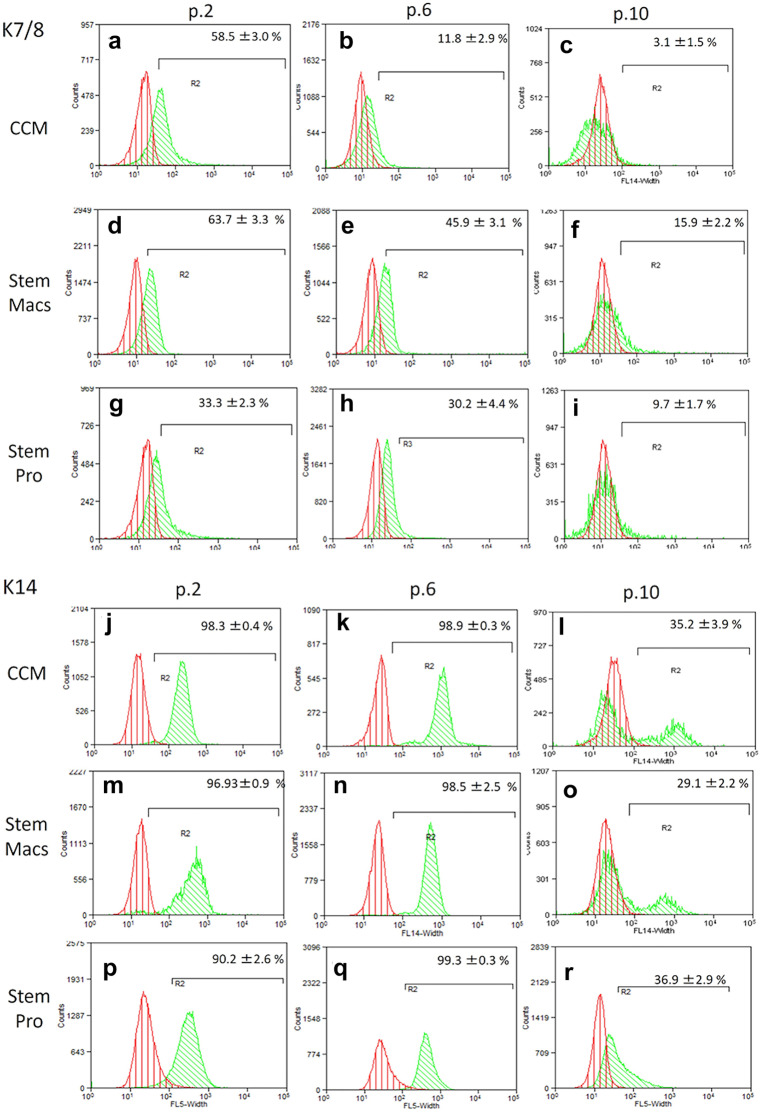
Flow cytometric analysis of **a**–**i** cytokeratins 7/8 and **j**–**r** cytokeratin 14 for each expansion medium at early, middle, and late passages. Values are mean (± SD) of three independent experiments of a single representative mSG-SCs

**Fig. 7:**
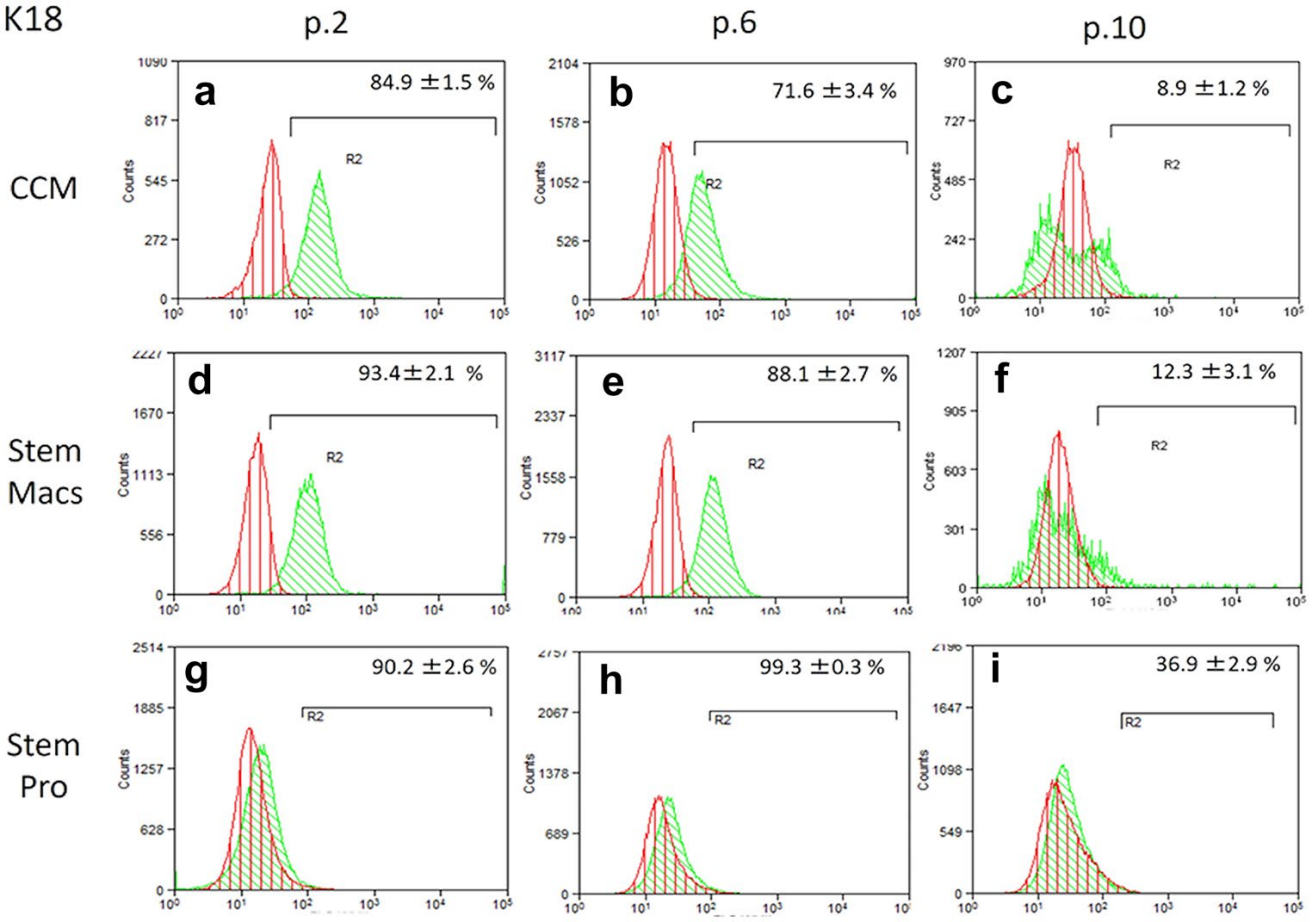
Flow cytometric analysis of **a**–**i** cytokeratin 18 for each expansion medium at early, middle, and late passages. Values are mean (± SD) of three independent experiments of a single representative mSG-SCs

The immunophenotype of mSG-SCs is studied in cultures with all three different culture media (CCM, StemMacs, StemPro) in early (p.2), middle (p.6), and late (p.10) passage for the expression of a range of stem cell membrane markers (CD90/Thy-1, CD73, CD146/MUC18, STRO-1, CD105, CD106, CD34, SSEA-4) and cytoplasmic epithelial cell markers (cytokeratins 7/8, 14, 18) by flow cytometry as shown in Figs. 2, 3, 4, 5, 6, and 7. The values ​​represent the mean of three different donors per culture media (3 biological replicates). Regarding the mesenchymal markers (CD90/Thy-1, CD73 (Fig. 2a–r) the results have shown, for mSG-SCs, an overexpression in all passages (p.2, 6, 10) and all different culture media. The marker CD146/MUC18 (Fig. 3a–i) showed an overexpression in the early passage (p.2) of all three different culture media and then gradually showed a significant decrease in mSG-SCs cultures in CCM (p.6: 74.2%, p.10: 24.3%), It has also been observed a moderate downregulation in StemMacs (p.6: 89.5%, p.10: 68.7% of cells), while StemPro maintained high overexpression levels (p.6: 91.2%, p.10: 88.1% of cells). The marker STRO-1 (Fig. 3j–r) showed an upregulation initially for the CCM (p.2: 84.9% of cells) which later decreases (p.6: 51.9, p.10: 35.6%). For the StemPro medium, the expression is initially moderate (p.2: 56.2%) and then decreases (p.6: 35.1%, p.10: 31.3%). Finally, for StemMacs, the expression of the marker is low in the beginning and then decreases even more (p.2: 22.3%, p.6: 15.7%, p.10: 13.5%). The marker CD105 (Fig. 4a–i) was observed to have an overexpression at early passages (p.2) in all three-culture media (CCM: 94.9%, StemMacs: 84.1%, StemPro: 99.1% of cells). Later, it was observed a downregulation for StemMacs (p.6: 74.1%, p.10: 53.9%), whereas a downregulation for CCM was observed only at late passages (p.10: 89.9%), and finally, the expression levels for StemPro were maintained at a high level (p.6: 93.3%, p.10: 97.3%) throughout the experiment. For marker CD106 (Fig. 4j–r), a consistent overexpression was observed for CCM (p.2: 92.1%, p.6: 95.3, p10: 85.9%); for StemMacs, there is an increase in expression for all passages (p.2: 86.7%, p.6: 91.5%, p.10: 93.3%), while on the contrary, there was a moderate downregulation for StemPro (p.2: 44.8, p.6: 38.9%, p.10: 12.4%). For the marker CD34 (Fig. 5a–i), a moderate expression was observed for CCM (p.2: 46.0%, p.6: 52.2%), with a notable decrease at a late passage (p.10: 7.8% of cells). For StemPro, a moderate expression was observed in all passages (p.2: 47.3%, p.6: 49.9%, p.10: 36.9%). For StemMacs, high expression in all passages was observed (p.2: 80.4%, p.6: 76.8%, p.10: 75.5%). For the marker SSEA-4 (Fig. 5j–r), the results have shown at early passage an upregulation for CCM (p.2: 85.3%), StemPro (p.2: 65.8%), and StemMacs (p.2: 70.9%). At later passages, it was observed that for StemPro, there was a maintenance of moderate expression (p.6: 55.3%, p.10: 47.3%), while a decrease on the expression for CCM (p 0.6: 69.1%, p.10: 66.9% of cells) and StemMacs (p.6: 57.3%, p.10: 49.9% of cells) was noticed. As far as the epithelial phenotype marker K7/8 (Fig. 6a–i) present in glandular secretory cells, the results have shown a moderate expression in all three-culture media in early passages (p.2: CCM: 58.5%, StemMacs: 63.7%, StemPro: 33.3%). However, a decrease was observed between, middle, and late passages for CCM (p.6: 11.8%, p.10: 3.1%), for StemMacs (p.6: 45.9%, p.10: 15.9% of cells), and for StemPro (p.6: 30.2%, p.10: 9.7%). Regarding the marker K14 (Fig. 6j–r), an overexpression profile was observed in the early and middle passages for all three-culture media, specifically CCM (p.2: 98.3%, p.6: 98.9%), StemMacs (p.2: 96.9%, p.6: 98.5%), and StemPro (p.2: 90.2%, p.6: 99.3%). However, during the late passage, a notable decrease was observed for all three different culture media, for example, CCM (p.10: 35.2% of cells), StemMacs (p.10–29.1% of cells), and StemPro (p.10: 36.9% of cells). Finally, regarding K18 (Fig. 7a–i), high expression levels were observed, similarly to K14, specifically, in early and middle passages for all three different culture media including CCM (p.2: 84.9%, p.6: 71.6%), StemMacs (p.2: 93.4%, p.6: 88.1%), and StemPro (p.2: 90.2%, p.6: 99.3%). A notable decrease in the expression of K18 was observed in all three different culture media at a late passage (p.10), with relative increase in StemPro, which indicated that it retained its epithelial characteristics to some extent (CCM: 8.9%, StemMacs: 12.3%, StemPro: 36.9%).

### Investigation of cellular aging (senescence) and telomere length of mSGCs

**Fig. 8:**
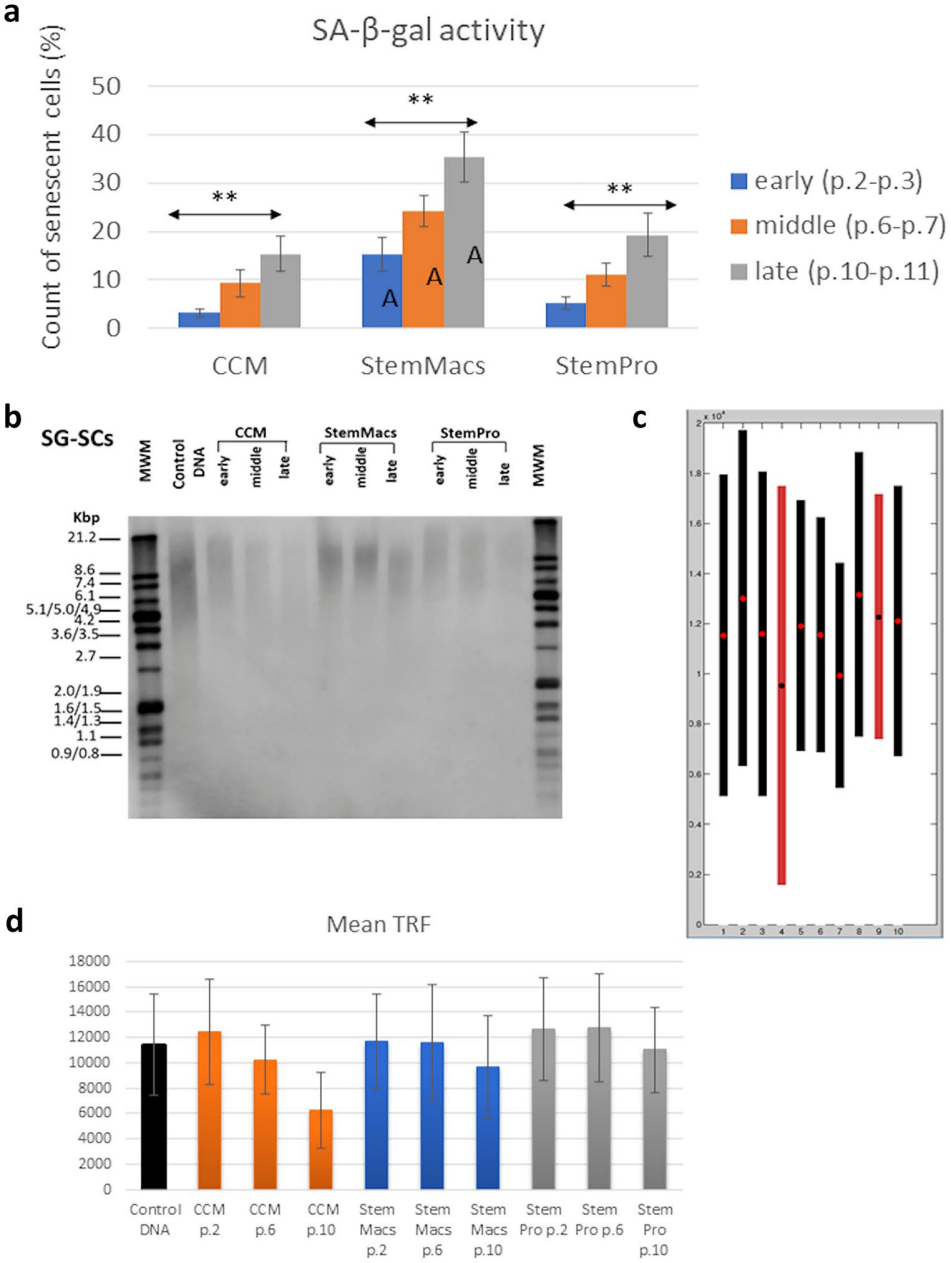
Study of cellular aging and telomere length of mSGCs. **a** Effect of three different culture media (CCM, StemMacs and StemPro) in mSG-SCs on the expression of β-galactosidase (SA-β-gal). The values ​​displayed are the averages (± constant deviations, SD) in mSG-SCs cultures (*n* = 3 cultures/culture medium, with repeat the biological experiments three times with two technical samples each). Asterisks indicate statistically significant differences (*p < 0.05; **p < 0.01) between the initial means and the later passages for each culture medium. The evaluation of the expression of β-galactosidase SA-β-gal showed a statistically significant increase between early, middle, and late passages (p.2–3, p.6–7, p.10–11) in all three different culture media but statistically significantly higher in all passages for StemMacs media (*p* < 0.01). **b** A representative southern blot from cells isolated from one donor and cultured in the three different culture media. The second line during the electro-display depicts a control DNA (control DNA) to confirm the correct hybridization and southern blot. Lines 1 and 12 are molecular weight indices (kbp). **c** The result of the analysis as obtained from the TeloTool tool. **d** Bar graphs which show the average TRF value (± constant deviation, SD) for each medium/passage. In summary, the length of telomeres was measured by terminal restriction fragment analysis (TRF) with southern blot, isolating the total genomic DNA isolated from cells in early (p.2–3), middle (p.6–7), and later (p.10–11) passages (Fig. 8b), and the analysis was obtained from the TeloTool tool (Fig. 8c). In Fig. 8d, the mean TRF is plotted showing a potential reduction of the telomere length in mSG-SCs for CCM at late passages, while for StemMacs and StemPro had no apparent difference in the aging of cells

These assays intended to examine whether the noticeable changes in immunophenotypic profiles, growth of cellular rate, and morphological characteristics were linked with cellular senescence indicative of unreliable proliferative function. Cellular senescence was investigated by evaluating the expression of β-galactosidase (SA-β-gal) and the length of chromosomes telomeres. As shown in (Fig. 8a), SA-β-gal shows a statistically significant increase between early, middle, and late passages (p.2–3, p.6–7, p.10–11) in all three different culture media (*p* < 0.01). This increase was statistically significantly higher in all passages for StemMacs media compared to the other two culture media CCM and StemPro (*p* < 0.01). Overall, StemMacs has shown the greatest number of aging cells and where gradually increased during subculturing at later passages followed by CCM and StemPro. These results coincide with the findings on cell proliferation (Fig. 1a) and the microscope images (Fig. 1(d–f'') that demonstrated a reduced number of cells with subsequent subculturing over different passages of mSG-SCs. Besides, the cellular senescence of mSG-SCs based on the length of chromosomes telomeres is investigated in all three different culture media, as shown in Fig. 8b–d. The length of telomeres was measured by terminal restriction fragment analysis (TRF), with southern blot, isolating the total genomic DNA from cells in early (p.2–3), middle (p.6–7), and later (p.10–11) passages (Fig. 8b). The analysis was obtained from the TeloTool tool (Fig. 8c). In Fig. 8d, the mean TRF is plotted showing a potential reduction of the telomere length in mSG-SCs for CCM at late passages, while for StemMacs and StemPro, no apparent differences in the aging of cells are observed (only a small reduction of telomere length in the late passages). This demonstrates that the cells maintained their stemness after several subculturing passages.

### Investigation of protein expression

**Fig. 9:**
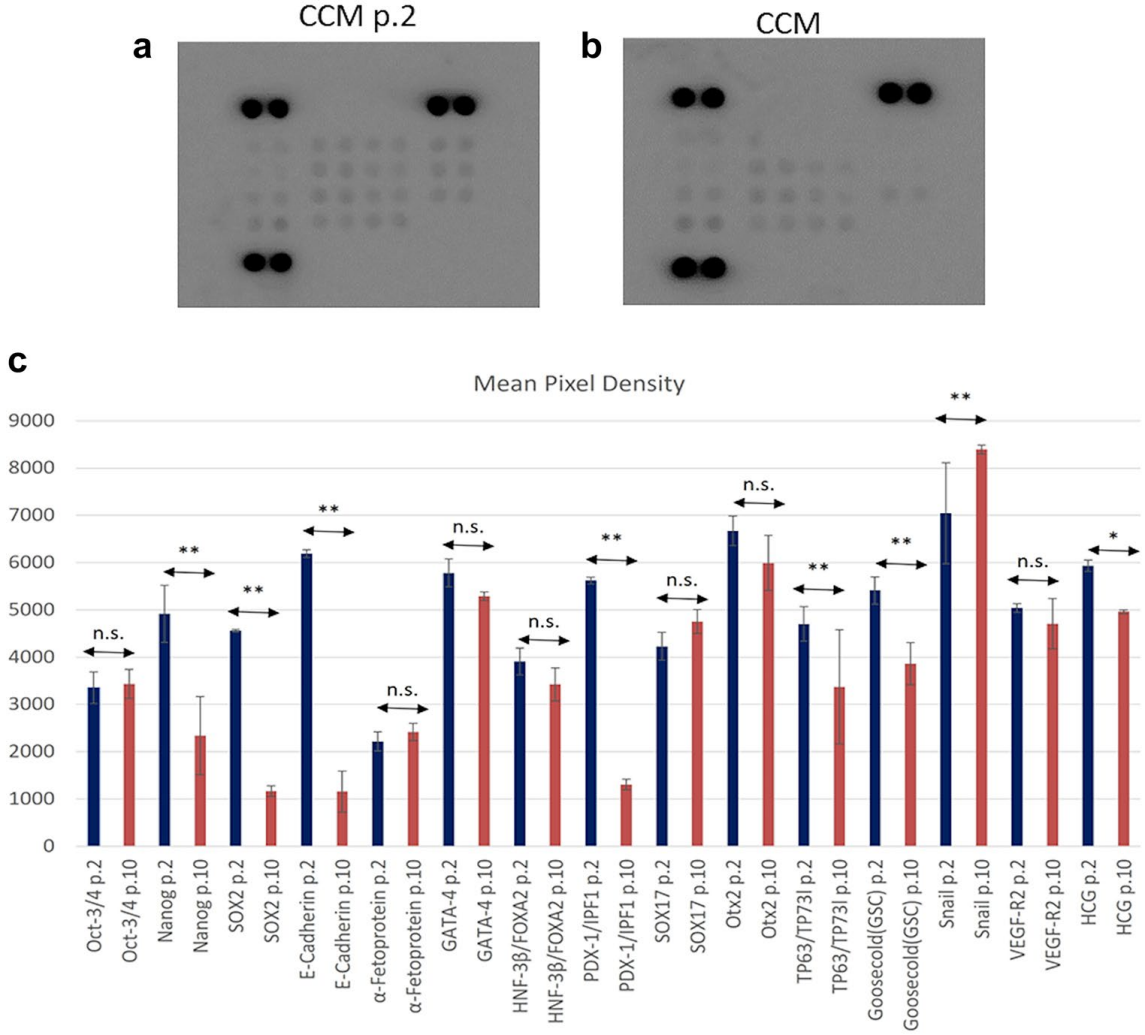
Proteomic analysis using proteomic assay kit which evaluates the expression of several stem cell markers, transcription factors, and epithelial markers for mSG-SCs cells cultured in CCM in early (p.2) and late (p.10) passages. **a** and **b** Expression levels are displayed with the corresponding positive dots on membranes. **c** Graph with mean values that have been normalized with the spots of each membrane converted to average values. The expression of several markers of pluripotency remained at high levels even after several passages (Fig. 9c). Oct-3/4, HNF-3β/FoxA2, and SOX17 showed no change in expression up to p.10, while Nanog (*p* < 0.01) and Goosecoid (GSC) (*p* < 0.01) demonstrated high expression in p.2 with a decrease in p.10. Also, transcription factors such as SOX2 (*p* < 0.01), GATA-4, PDX-1/IPF1 (*p* < 0.01), Otx2, VEGF R2/KDR/Flk-1, HCG (*p* < 0.05), and α-Fetoprotein (AFP) expressed in stem cells. In addition, E-cadherin (*p* < 0.01), an exclusive epithelial cell marker, was overexpressed in p.2 and was decreased in p.10. In contrast Snail (*p* < 0.01), a stem cell marker which also suppresses E-cadherin was increased from p.2 to p.10. Oncoprotein p63/TP73L/TP63 (*p* < 0.01) was downregulated indicating a decrease in the proliferative potential of the mSG-SCs during passaging

To complete the characterization of mSG-SCs cultured with CCM in p.2 and p.10 using different markers, as shown in Fig. 9a–c, a proteomic assay kit is used highlighting passages p.2 and p.10. The relative expression levels are presented with values ​​that have been normalized with the corresponding dots (Fig. 9a, b) of each membrane converted to mean values (Fig. 9c). Based on the findings, it can be noted that the expression of several markers of pluripotency remained at high levels even after several passages (Fig. 9c). Among these markers, Oct-3/4, HNF-3β/FoxA2, and SOX17 showed no change up to p.10, while Nanog (*p* < 0.01) and Goosecoid (GSC) (*p* < 0.01) demonstrated high expression in p.2 with a decrease in p.10. Also, the expression of transcription factors such as SOX2 (*p* < 0.01), GATA-4, PDX-1/IPF1 (*p* < 0.01), Otx2, VEGF R2/KDR/Flk-1, HCG (*p* < 0.05), and α-Fetoprotein (AFP) was notable in stem cells. In addition, there was an elevated expression of E-cadherin (a cell adhesion molecule exclusive for epithelial cells) in mSG-SCs of p.2 with significant decrease in p.10 (*p* < 0.01). On the other hand, Snail which suppresses E-cadherin and is a marker of stem cells was overexpressed from at p.2 to p.10, (*p* < 0.01). Finally, oncoprotein p63/TP73L/TP63 (*p* < 0.01) was downregulated indicating a decrease in the proliferative potential of the mSG-SCs during passaging.

### Expression of lineage-specific markers

**Fig. 10:**
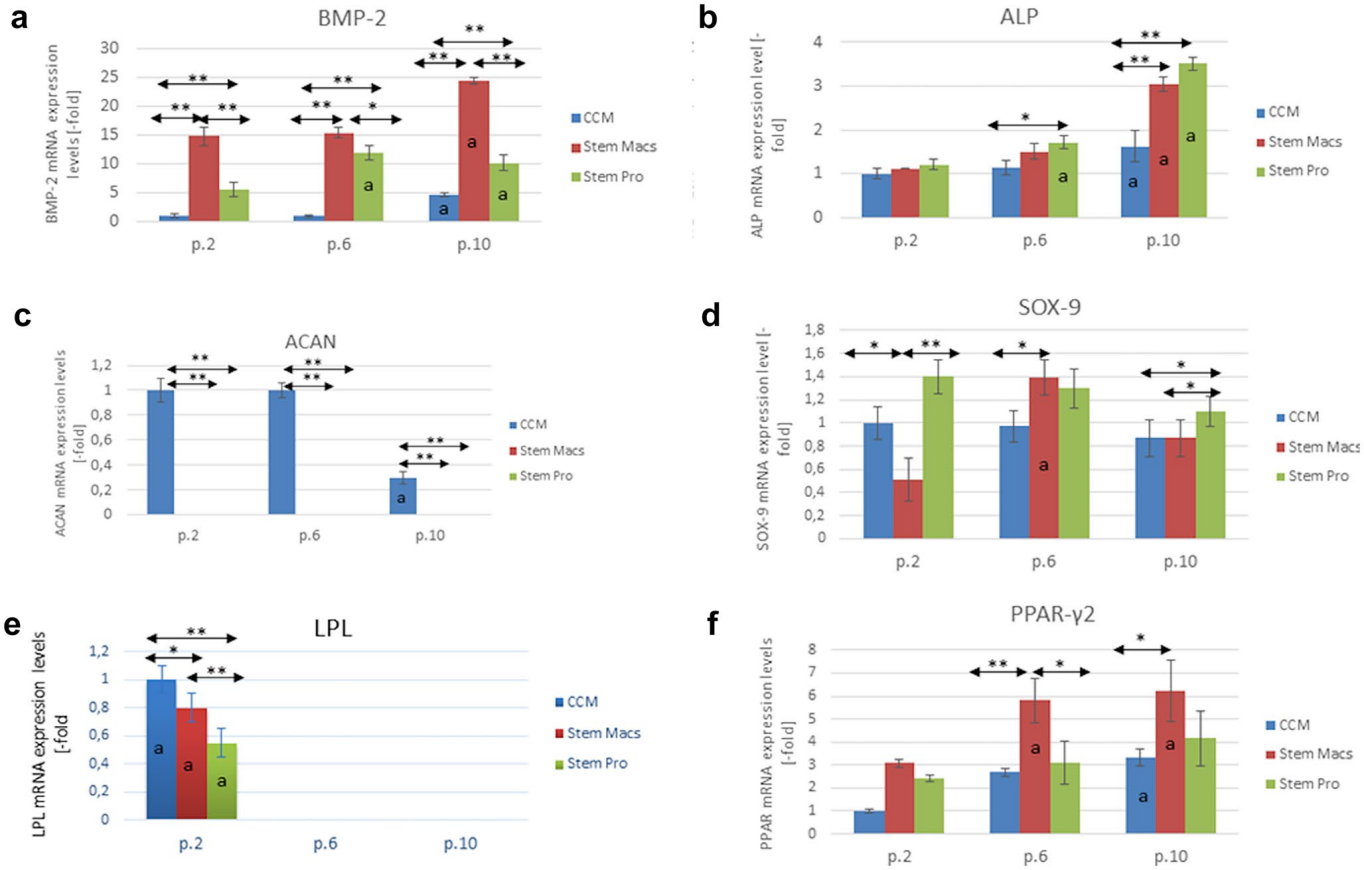
**a**–**f** Evaluation by real-time PCR of expression of markers for osteogenic (ALP, BMP-2), chondrogenic (SOX-9, ACAN), and adipogenic (PPAR-γ2, LPL) differentiation in mSG-SCs after several subculturing in three different media (CCM, StemMacs, and StemPro). Mean values ​​are recorded (± constant deviations, SD) (*n* = 3/culture medium, repeating biological experiments three times with two technical samples each). Asterisks indicate the statistically significant differences (*p < 0.05; **p < 0.01) between the individual culture media in each passage, while small a indicates the differences from the original expression (p.2) in each medium (i.e., at p.6 and p.10 relative to p.2)

The results for the potential for osteogenic, chondrogenic, and adipogenic differentiation vary significantly among three different culture media (CCM, StemMacs, and StemPro), as shown in Fig. 10a–f. Specifically, the osteogenic marker BMP-2 (Fig. 10a) was expressed at particularly low levels in mSGSCs cultured with CCM at p.2 and p.6, with a slight increase to p.10 (*p* < 0.05). For StemPro, the expression of BMP-2 showed a statistically significant increase already from p.6 (*p* < 0.01), while in StemMacs, the baseline of BMP-2 was already increased compared to the other two culture media in p.2 (*p* < 0.01) and showed a further statistically significant increase up to p.10 (*p* < 0.01). In Fig. 10b, the ALP was gradually increased in all passages (p.2, p.6, p.10) for all three different culture media (*p* < 0.05 for CCM and *p* < 0.01 for StemMacs and StemPro). For StemPro, it was observed a statistically significant difference in expression compared to CCM at p.6 (*p* < 0.05) and p.10 (*p* < 0.01). Similarly, for StemMacs, ALP expression demonstrated moderate expression values ​​between CCM (lower) and StemPro (higher). Especially in p.10, ALP expression was statistically higher compared to CCM (*p* < 0.01). For ACAN (Fig. 10c), it was not detectable in all passages (p.2, p.6, p.10) for StemMacs and StemPro, while there was a positive expression at p.2 and p.6 with a significant decrease at p.10 (*p* < 0.01) for CCM. For the marker SOX-9 (Fig. 10d), it was observed for StemPro (p.2) a statistically significant upregulation in the expression compared to StemMacs (*p* < 0.01) and higher in relation to CCM (*p* < 0.05). At p.6, SOX-9 demonstrated an increase in the expression for StemMacs compared to p.2 and even statistically higher compared to CCM (*p* < 0.05) and StemPro. Finally at p.10, there was an overexpression of SOX-9 for StemPro compared to CCM/StemMacs (*p* < 0.05).

LPL expression was observed only at the early passages (Fig. 10e) (p.2) for all three different types of media. There was an apparent upregulation for CCM compared to StemMacs (*p* < 0.05) and StemPro (*p* < 0.01). In addition, there was a statistically significant increase in LPL expression for StemMacs compared to StemPro (*p* < 0.01). Ultimately, the transcription factor PPAR-γ2 (Fig. 10f) was upregulated in all passages (p.2, p.6, p.10) of all three different culture media, but an apparent increase was noted for StemPro and StemMacs. Specifically at p.6, the PPAR-γ2 expression was greater for StemMacs compared to CCM (*p* < 0.01) and StemPro (*p* < 0.05). Finally, at p.10, the expression of PPAR-γ2 for StemMacs was statistically higher compared to CCM (*p* < 0.05, and even greater for StemPro.

### Osteogenic and chondrogenic differentiation potential

**Fig. 11:**
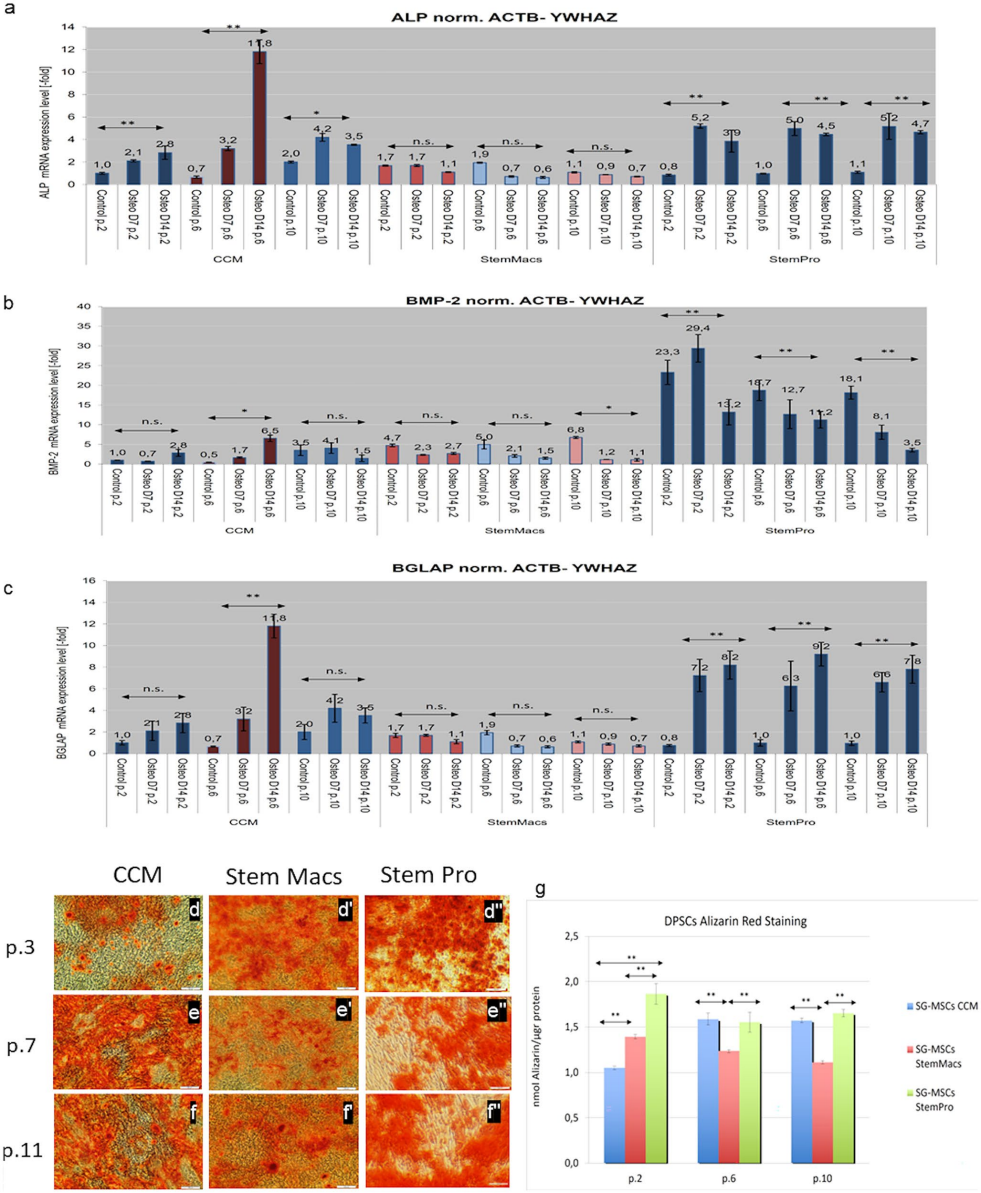
**a**–**c** Real-time PCR analysis of the expression of osteogenic marker genes (ALP, BMP-2, BGLAP) after corresponding induction mSG-SCs from passages p.2 (early), p.6 (intermediate), and p.10 (late) grown in the three different media (CCM, StemMacs, and StemPro). Values ​​are mean (± SD) of three (*n* = 3) different experiments with two repetitions each. The asterisks above the horizontal double arrows indicate the statistically significant differences (*p < 0.05; **p < 0.01; ns = non-significant) in each passage for each culture medium (CCM, StemMacs, StemPro), during induction (osteogenic/coarse) for 7 and 14 days (D0, D7, and D14). **d**–**f**'' Histochemical staining with alizarin red in three different cultures in CCM, StemPro, and StemMacs. In initial (p.2), medium (p.6), and final (p.10) stage passage of the experiment. **g** Spectrophotometric evaluation of in vitro bioremediation (measurement of bound alizarin) expressed as mean mmol alizarin from three independent experiments with three replications each, in the culture of mSG-SCs developed in principle in one of the three different media, namely CCM, StemPro, and StemMacs and then in each passage (initial p.3, mean p.7, and final p.11) induction of differentiation with a special culture medium for 21 days. The statistics comparisons between passages and culture media were made by analysis two-way ANOVA and Tukey’s post-hoc tests

**Fig. 12:**
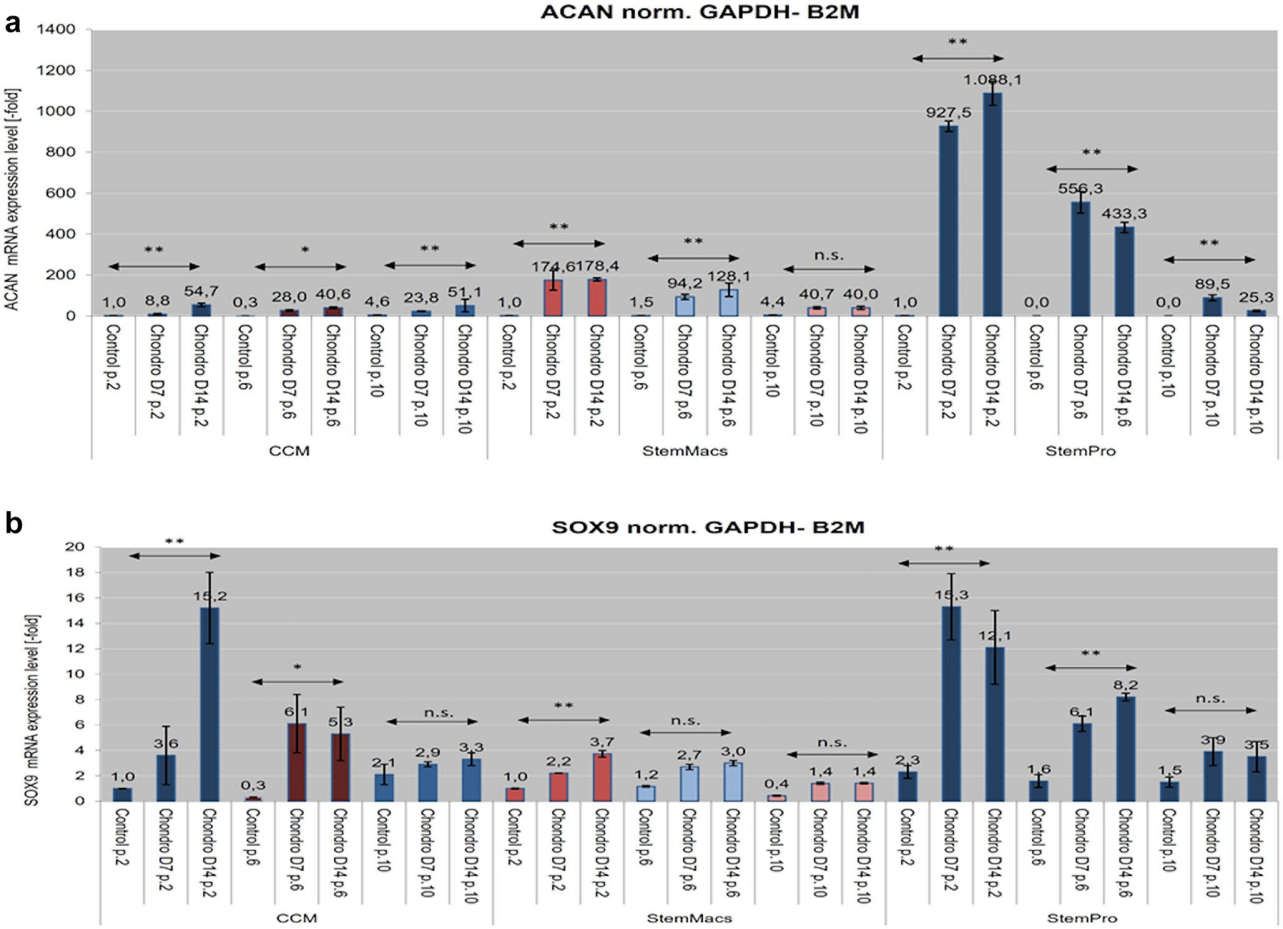
Real-time PCR analysis of the expression of chondrogenic differentiation markers, including **a** ACAN and **b** SOX-9 after corresponding induction mSG-SCs from passages p.2 (early), p.6 (intermediate),and p.10 (late) grown in the three different media (CCM, StemMacs and StemPro). Values ​​are mean (± SD) of three (*n* = 3) different experiments with two repetitions each. The asterisks above the horizontal double arrows indicate the statistically significant differences (*p < 0.05; **p < 0.01; ns = non-significant) in each passage for each culture medium (CCM, StemMacs, StemPro), during induction (osteogenic/coarse) for 7 and 14 days (D0, D7, and D14)

These assays intended to elucidate if there were considerable variations in gene expression patterns through extended growth, as well as indicate changes of the differentiation potential of mSG-SCs at early, middle, and late passages. The evaluation of the differentiation potential towards osteogenic and chondrogenic phenotype is crucial for the determination of “stemness” of the cultured cell populations under variable microenvironmental conditions. Several osteogenic and chondrogenic markers are evaluated in mSG-SCs cultures (all three different type of media) at different passages (p.2, p.6, and p.10) in a time response experiment (0, 7, and 14 days), as shown in Fig. 11a–g for osteogenic and Fig. 12a, b for chondrogenic, respectively. For osteogenic differentiation, the following markers ALP, BMP-2, and BGLAP were investigated. In Fig. 11a, CCM demonstrates an upregulation in expression for ALP from day 0 to day 14 in all passages (*p* < 0.01 for p.2 and p.6 and *p* < 0.05 for p.10 with maximum expression on day 7 for the latter). For StemMacs, no change in baseline expression was observed at all passages. Also, for StemPro, there was a statistically significant increase in ALP expression (*p* < 0.01 for all passages) at day 7. In Fig. 11b, CCM demonstrates a slight increase in expression for BMP-2 between day 0 to 7th and 14th at passage 2. In p.6 the low expression level on day 0 and day 7 was followed by a statistically significant increase at day 14 (*p* < 0.05). Finally, for p.10, there was no statistically significant changes between days 0, 7, and 14. For StemMacs, p.2 and p.6 demonstrated similar expression pattern for BMP-2 at days 0, 7, and 14. Similar pattern was observed in p.10 with statistically significant decrease at day 0 to days 7 and 14 (*p* < 0.05). For StemPro, in p.2, there was increase in the expression at day 0 to day 7, and then, there was a statistically significant decrease up to day 14 (*p* < 0.01). In p6 and p.10, there was a statistically significant increase in the expression of BMP-2 between day 0 to 7 and 14 (*p* < 0.01). In Fig. 11c, CCM reveals a stable expression for BGLAP between days 0, 7, and 14 in p.2. However, in p.6, there was a statistically significant increase at day 14 (*p* < 0.01). Finally in p.10, there was a non-significant increase at day 7 and later a decrease at day 14. For StemMacs, there was no significant expression for BGLAP in all passages. For StemPro, there was a notable increase in BGLAP expression at days 7 and 14 for all three passages (p.2, p.6, p.10) (*p* < 0.01). To further evaluate the osteogenic differentiation, Alzarin staining (AR-S-based assay) is used as shown in Fig. 11d–g. Specifically, an in vitro calcification (biomineralization) was observed in all three types of media and in all passages (p.3, p.7, and p.11). There is an apparent increase in the biomineralization for CCM compared to StemPro and StemMacs as shown in Fig. 11d–f''. Quantification of the Alzarin red staining is performed as shown in Fig. 11g. It showed a statistically significant in vitro biomineralization of StemPro and StemMacs compared to CCM (StemPro > CCM, *p* < 0.01, and StemMacs > CCM, *p* < 0.01) at early passages. In p.6, there was an increase in in vitro biomineralization for CCM and in contrast a decrease for StemPro and StemMacs. Finally, in p.10, there was a decrease of the in vitro biomineralization for StemMacs, whereas for CCM, there was no apparent difference. Also, there was a statistically significant increase for CMM (*p* < 0.01) and StemPro (*p* < 0.01) compared to StemMacs. As far as chondrogenic differentiation is concern, several markers were evaluated such as ACAN and SOX-9. In Fig. 12a, for CCM, there is a relatively low expression for ACAN in p.2, p.6, and p.10 characterized for a gradual increase between days 0–7–14. Also, there was a statistically significant increase between passages (p.2, *p* < 0.01, p.6, *p* < 0.05, p.10, *p* < 0.01). For StemMacs, there was a statistically significant increase in the expression in p.2 at day 7 and day 14 compared to day 0 (*p* < 0.01). Similarly in p.6 there was a gradual increase at day 7 and day 14 compared to day 0 (*p* < 0.01). Finally, in p.10, there was no statistically significant expression between days 7 and 14 compared to day 0, indicating the inability of mSGCs to differentiate towards a chondrogenic phenotype after several subculturing. For StemPro, there was an apparent expression of ACAN in all passages. Specifically, in p.2, there was a gradual significant increase between days 0 and 7 and day 14 (*p* < 0.01). In p.6, there was a statistically significant increase between day 0 and day 7 (*p* < 0.01) and then a slight decrease at day 14. Finally, in p.10, there was a significant increase in ACAN expression at day 7 and then a decrease at day 14 (*p* < 0.01). In Fig. 12b, for CCM, there is a statistically significant increase in the SOX-9 expression at day 7 and even higher at day 14 (*p* < 0.01) in p.2. However, in p.6, there was an increase in expression at day 7 followed by a stabilization in the expression at day 14 (*p* < 0.05). Finally, in p.10, there was a non-statistically significant increase between days 0 and 7 and day 14. For StemMacs, there was a non-statistically significant increase in the expression in all passages (p.2, p.6, and p.10) and at all days (0, 7, and 14). For StemPro, there was a statistically significant increase in the expression in p.2 at day 7 followed by a decrease at day 14 compared to day 0 (*p* < 0.01). In p.6, there was a statistically significant increase between days 0 and 7 and day 14 (*p* < 0.01). Finally, in p.10, there was a non-statistically significant increase in the expression at day 7.

## Discussion

The salivary glands’ pathology leads to a negative outcome in patients’ QoL, which makes the need for rehabilitation crucial (Schwarz and Rotter 2012; Vissink et al. 2003; FOX 1998). Stem cells have been isolated and characterized immunophenotypically from major and oral mucosal minor salivary glands in human and animals with or without prior induction of various agents. However, especially for the minor salivary glands, the information is very limited and contradictory (Weng et al. 2019; van Luijk et al. 2015; Aure et al. 2015; Mitroulia et al. 2019; Lu et al. 2015). Stem cell culture systems have been developed in two-dimensional and three-dimensional cultures, and advances in the field of tissue engineering of salivary glands enable the development of new methods to regenerate the damaged gland (Zhang et al. 2017; May et al. 2018; Okumura et al. 2003). Salivary glands stem cells include all the general characteristics of stem cells (stemness), for example, the high proliferative rate and the ability of self-renewal, colony-forming units (CFUs), and the potency for trilineage differentiation (chondrogenic, adipogenic, and osteogenic) (Kishi et al. 2006; Gorjup et al. 2009).

The isolation of minor salivary gland stem cells from the lips is a source of stem cells easily accessible and safe as these glands are mainly distributed superficially in the mucosal side of the lips. It is a minimally invasive technique causing no trauma. It is important to mention that there are two basic approaches regarding the microscopic location of salivary gland stem cells. First, stem cells (of epithelial origin) may be found throughout the acinar and ductal epithelium (basal cells) or found at the epithelial lining of a specific type of ducts named intercalated ducts (Kagami et al. 2008). Secondly, the mesenchymal stem cells (MSCs) are in the surrounding-supportive connective tissue of the salivary epithelial structures (acini-ducts) (Coppes and Stokman 2011). Several research studies have been designed for the clinical application of stem cells and their products in the effort for tissue regeneration of salivary glands (Emmerson and Knox 2018). In this context, an important parameter to be explored is the type of culture medium used, as this directly affects the biological characteristics of the isolated populations. From the literature review, it appears that the present research is the first, on comparative isolation and long-term stem cell proliferation of salivary glands, with extensive characterization and analysis with the use of three different culture media: specifically, two of them were prepared under cGMP conditions compared to a more conventional culture medium based on fetal bovine serum. As mentioned previously, the main point of these studies is to investigate how a culture medium with no FBS supplemented could meet the requirements for cGMP culture (in time, quality, and quantity). These cGMP requirements are: these cultures to produce large yields of cells that are able to survive, to maintain their stem cell characteristics, and of course to be safe for clinical application (Bakopoulou et al. 2017). The necessity to conduct the study was related to the investigation of different tissue culture conditions that may affect the characteristic properties of stem cells such as proliferation ratio, aging, immunophenotype, and differentiation capacity. One solution proposed for the culture of stem cells under cGMP conditions is the use of a medium containing serum of human origin (autologous or not). However, difficulties associated with the high heterogeneity of autologous human serum and the limited commercial availability of human heterologous serum pose significant limitations in their use for long-term cell cultures (Yu et al. 2010; Kim et al. 2012; Ferro et al. 2012; Fekete et al. 2012). Also, various supplements and changes to currently used culture media have given vague results (Sotiropoulou et al. 2006; Gharibi and Hughes 2012; Kumar et al. 2010).

Previous studies are in agreement with our results (Kwak et al. 2018), where mSGCs in culture expressed all the stem cell characteristics (embryonic markers Nanog, Oct3/4, SSEA etc.) and mixed immunophenotype between mesenchymal type (expression of CD90/Thy-1, CD73, CD146-1, CD105, CD106, CD34) and glandular epithelial origin (keratin expression, E-cadherin). Furthermore, an initially morphologically heterogeneous population consisting of ovoid-spheroidal (epithelial-phenotype) together with elongated, spindle-shaped cells (mesenchymal phenotype) was observed. In the present study, this mixed cell population has a significant proliferation rate, which initially did not differ between the three-culture media investigated. However, during the time course, cellular morphological homogeneity appears with the mesenchymal phenotype dominating the cell culture. This rather homogeneous population combines mesenchymal and epithelial (mixed) immunophenotype, as shown by the expression of mesenchymal markers, as well as keratin markers even after a prolonged period of culture. Keratin 14 is expressed in adult myoepithelial cells, but in addition, according to Adhikari et al., CKs 14 and 18 could also be found in acinar cells during acinar cell development (Adhikari et al. 2018). We detected keratin 14 because Keratin-14^+^ (K14^+^) cells have gained increasing attention given that they are stem/progenitor cells that are actively involved in salivary gland development, homeostasis maintenance, and regeneration following injury (Zhang et al. 2022). Furthermore, Kusama et al. mentioned that CK18 was detected in the cytoplasm of acinar/ductal cells in normal human salivary glands (Kusama et al. 2000). In fact, we have already shown in our previous manuscript (Andreadis et al. 2014) the presence of another member of Aquaporins (AQ1) in the minor salivary gland stem cells. AQ1 normally is expressed in *terminal bud stage* at the apicolateral membrane of acinar cells (de Paula et al. 2017). This mixed stem cell population seems to have the potential for differentiation into different cell lines. Interestingly, the proliferation rate in a late passage was reduced with StemMacs culture medium compared to the other two media which indicated that StemMacs might accelerate telomere lose. On the contrary, the cell population expansion in StemPro showed a remarkable increase in the proliferation rate with more prominent stem cell characteristics during prolong cultivation. This result was confirmed with increased immunophenotyping expression of these cells with the corresponding stem cell markers, which indicated maintenance of stemness under the specific cultivation process (Lin et al. 2011).

Specifically, it was found that cells cultured with StemPro medium showed higher expression in the long run for stem cell markers CD146, Stro-1, and CD105 while the markers CD106 and CD34 had higher expression in cell culture with StemMacs medium. The keratins evaluated in this study had a specific expression pattern. Specifically, Keratin CK7 showed high expression in stem cells in the three different culture media; however, during subculturing, there was a decrease in the expression of CK7 especially for CCM and less for StemPro and StemMacs. This maintenance of keratin expression for StemPro and StemMacs was also present for CK14. This suggests an advantage of the cultured media especially StemPro for maintaining the stemness of a mixed population that has an immunophenotypic of both stem cell and epithelial origin during prolong cultivation. In addition, one of the major findings of this study was the confirmation of co-expressed markers of mesenchymal and epithelial origin. Using special proteomics kit, the increased expression of E-cadherin in the salivary stem cells at an early passage (p.2) followed by a significant decrease at a later passage (p.10), in combination with simultaneous increase of Snail expression during the same passages (p.2–p.10), confirmed the morphology of the cell populations but also the profile expression of the keratins. This observation coincides with the fact that initially the stem cells of the salivary glands co-exist with an epithelial population of glandular cells that over time they are replaced by mesenchymal stem cells which eventually become the majority.

The increase in the oncoproteins p63/TP73L/TP63 indicated the extensive transition of these cells to a mesenchymal stem cell population with strong proliferative potential. Further study is needed in cell cultures with culture media without FBS as in the proteomic technique that was used in this study; we were limited to culturing salivary gland stem cells in CCM as control. Criterion for the efficiency of a stem cell culture system after subculturing in prolonged incubation periods is the aging (cellular senescence). The evaluation of aging of stem cells is important since several morphological, metabolic changes occur. Furthermore, it is noticed secretion of inflammatory factors (senescence-associated secretory phenotype, SASP) with a negative impact on the possibility of their clinical application (Schulz et al. 2012). The finding of this unavoidable aging that was first evaluated for salivary gland stem cells in the present study was less evident in StemPro based on the expression of SA-β-gal and more on StemMacs. Respectively, there is a tendency for progressive reduction of the telomere length in salivary gland stem cells cultured in CCM especially in late passages, while in the other culture media (StemMacs, StemPro) and especially in StemPro, there was no significant reduction in telomere length, which indicated the maintenance of their stem cell properties after prolonged culture. Telomere length is a primary indicator of MSC replicative (telomere-associated) senescence (Wagner Wolfgang and Horn 2008) characterized by progressive loss of the telomeric TTAGGG repeats (Campisi and d’Adda di Fagagna 2007). On the other hand, it has been supported that SA-β-gal-activity, although associated, is neither causative nor specific for senescence. Instead, it should be interpreted in combination with other biomarkers (Batsali et al. 2017) However, given the expression of β-galactosidase has been mostly associated with a non-proliferative state, the increased proportion of beta-gal-positive cells in StemMacs, combined with the findings of the telomere length assay, provides robust evidence that this medium cannot maintain stemness of mSGCs and may be implicated in the disparate growth characteristics observed for different expansion systems. Besides, in line with the discrepancy of the beta-galactosidase and telomere length assay observed for the CCM medium, a previous study (Mason et al. 2013) showed no significant shortening (< 1 kbp) in mean telomere length of aBMMSCs at early (p.1–4; PD < 25) and middle (p.5–8; PD < 50) passages, while at the same time significantly greater proportions of SA-β-gal-positive cells became evident over time.

The present study also evaluated the differentiation capacity of stem cells at different time points with all three-culture media, and it was found a tendency of stem cells to prefer an osteogenic differentiation in the presence of StemPro.

## Conclusions

In this study, the properties and characteristics of mixed stem cell populations, resulting from minor salivary gland stem cells of the lower lip mucosa, have been extensively studied and characterized. After analysis of their immunophenotype, it appeared that the proliferating cells are a mixed population that have stem cell characteristics (expression of embryonic and mesenchymal markers) and epithelial characteristics (expression of specific keratins and E-Cadherin) of glandular epithelium. In addition, the analysis of our results showed the strong potential of these cells for trilineage differentiation. This needs further investigation because the potential of multilineage differentiation of mSGSCs to other glandular tissues, for example, pancreas, liver, kidneys, or thyroid among others, could be of therapeutic value for tissue regeneration and clinical applications. In the second part of the study, the characteristics of mSGSCs, in terms of maintaining their stem cell properties (stemness), during prolonged incubation periods had been investigated, as it represents a critical aspect for regenerative medicine applications. These technical characteristics involved the isolation, proliferation, and evaluation of aging (cellular senescence) with different culture media (StemPro and StemMac) to meet cGMP requirements towards a safe and effective future stem clinical application of mSGSCs according to international guidelines. Based on the results, it appeared that the isolated mSGSCs have a good proliferation rate in serum-free media (StemPro, StemMACS) compared to conventional serum medium (CCM) and maintained their stem cell properties (stemness) and their differentiation capacity after prolonged incubation periods (multiple subculturing) especially in the StemPro medium. Future approaches may include the investigation of the integration of additional factors in this type of culture media that may enhance media’s effectiveness substituting the absence of serum with a—in parallel—further investigation by means of pre-clinical studies with experimental animal models to recapitulate the in vivo analogue.

## Data Availability

The datasets supporting the results of this article are included within the article.
